# Can remote ischemic preconditioning counteract the renal functional deterioration attributable to partial nephrectomy under warm ischemia? Results of an animal study

**DOI:** 10.1186/s12882-021-02359-1

**Published:** 2021-07-16

**Authors:** Tuna Till Mut, Ömer Acar, Ayse Armutlu, Said Incir, Annemarie Uhlig, Lale A. Ertuglu, Melis Özel, Ali Cihan Taskin, Dilek Ertoy Baydar, Mehmet Kanbay, Tarık Esen

**Affiliations:** 1 Department of Urology, University Medical Center Göttingen, Robert-Koch Straße 40, 37075 Göttingen, Germany; 2 Department of Urology, Koc University School of Medicine, Istanbul, Turkey; 3 Department of Pathology, Koc University School of Medicine, Istanbul, Turkey; 4 Department of Biochemistry, Koc University Hospital, Istanbul, Turkey; 5 Koc University School of Medicine, Istanbul, Turkey; 6 Koc University, Center for Translational Medicine (KUTTAM), Istanbul, Turkey; 7 Department of Internal Medicine, Division of Nephrology, Koc University School of Medicine, Istanbul, Turkey; 8 Department of Urology, VKF American Hospital, Istanbul, Turkey

**Keywords:** Kidney, Ischemia, Function, Preconditioning, Remote, Biomarker

## Abstract

**Background:**

To investigate if remote ischemic preconditioning (RIPC) can offer any renoprotective value by counteracting the deleterious effect of partial nephrectomy (PN) under warm ischemia on renal function.

**Methods:**

Four groups, each with 5 Wistar albino rats, were constructed; RIPC + PN, PN, RIPC and sham. Right nephrectomy was performed to constitute a solitary kidney model. RIPC denoted sequential clamping/declamping of the femoral artery/vein complex. PN was performed under warm-ischemia following RIPC. Blood samples were collected on multiple occasions until euthanasia on day 7.

Immunoassays were conducted to measure the serum and tissues levels of kidney injury markers. Kidneys were examined histologically and morphometric analyzes were performed using digital scanning.

**Results:**

IL-33 levels did not differ significantly between the groups. Serum levels of KIM-1, NGAL, and aldose reductase in RIPC + PN, PN and RIPC groups were significantly lower than that of sham group. Tissue biomarker levels were similar across groups.

The observed trend in mean necrosis area of PN group was higher than that of RIPC + PN group (*p* > 0.05). The transitional zone between necrosis and healthy tissue showed a trend towards increasing width in the rats subjected to RIPC before PN vs. those who underwent PN without RIPC (p > 0.05).

**Conclusion:**

RIPC failed to counteract the renal functional consequences of PN under warm ischemia in a solitary kidney animal model. The supportive but marginal histological findings in favor of RIPC’s renoprotective potential were not supplemented with the changes in serum and tissue biomarker levels.

## Background

Partial nephrectomy (PN) is the preferred surgical treatment modality for localized renal tumors [1]. PN is commonly performed under warm-ischemic conditions in an effort to provide a relatively “bloodless” surgical field which will facilitate complete excision of the mass and subsequent reconstruction of the tumor bed. However, temporary interruption of the renal blood flow can be a predisposing factor for de-novo chronic kidney insufficiency [2]. For this reason, it is recommended that the ischemic duration should be limited to 20–30 min [3].

Ischemic preconditioning (IPC) is an innate tissue adaptation mechanism whereby repeated brief ischemia episodes trigger local and/or remote organ protection against succeeding exposure to the same or other type of injury [4, 5]. Local IPC has been mainly investigated in animal studies with varying degrees of success and owing to the technical limitations related to the extrapolation of local preconditioning methodology to clinical setting, further studies were concentrated around its remote counterpart [6, 7].

Remote ischemic preconditioning (RIPC) has been postulated as a measure that might potentially reverse or minimize the ischemia/reperfusion-related functional insult involving various organs. It has been defined as repeated brief ischemia episodes at a remote site before an anticipated ischemia/reperfusion injury of the target organ. An IPC regimen applied only 5 min prior to warm ischemia has been shown to offer significant protection against renal functional impairment in rats [8]. However, no consensus exists regarding the optimal durations of the preconditioning protocol itself and the latent period after which IPC exerts its beneficial effect. Several studies focused on the renoprotective effect of RIPC, which would theoretically enable prolongation of the ischemic duration within the context of PN, especially while treating renal masses with complex morphometric features [6]. However, relying on markers with poor specificity (such as serum creatinine level) while assessing the renal functional changes attributable to RIPC and/or performing the experiment in the presence of two functioning kidneys plagued the interpretation and utility of their findings potentially in favor of RIPC [9–11].

Several novel biomarkers of acute kidney injury (AKI) have been introduced for diagnostic and predictive purposes and these might be of greater value while testing how RIPC influences renal function, as subtle changes would remain undetectable by biomarkers with higher signaling threshold [12]. KIM-1 and NGAL are inducible biomarkers, with significantly increased levels as a direct response to nephron damage [13, 14]. KIM-1, in particular, is activated after epithelial injury and is thought to play a role in tubular regeneration process. It has been demonstrated that NGAL concentrations, in both serum and urine, increased promptly, within hours of ischemia and nephrotoxic insult [15]. IL-33 is an acute inflammatory marker and it has been shown that anti-inflammatory drugs could inhibit renal damage by reducing the expression of IL-33, which supports its value as a marker of AKI [16]. Ischemia driven aldose reductase overexpression in renal tissue has been demonstrated to be an important step in the pathogenesis of CKD [17].

In light of these data, we aimed to assess the impact of RIPC on renal functional preservation in a solitary kidney PN model by incorporating the data gathered by more sensitive and specific biomarkers of kidney injury in addition to histological analysis.

## Methods

The study was approved by Local Ethics Committee for Animal Experiments of Koc University (Approval no: 2015–19). The animals were kept in Koç University Animal Research Facility of Center for Translational Medicine (KUTTAM) under 12 h light–12 h dark cycle, and a diet of commercial pellet food ad libitum and automatic water containers were provided. Twenty-Five Wistar albino rats were included. The first 5 animals were used for the pilot study in which procedural steps were tested. The remaining 20 animals were divided into 4 groups: sham, RIPC, RIPC + PN, and PN.

### Surgical procedures

**Fig. 1:**
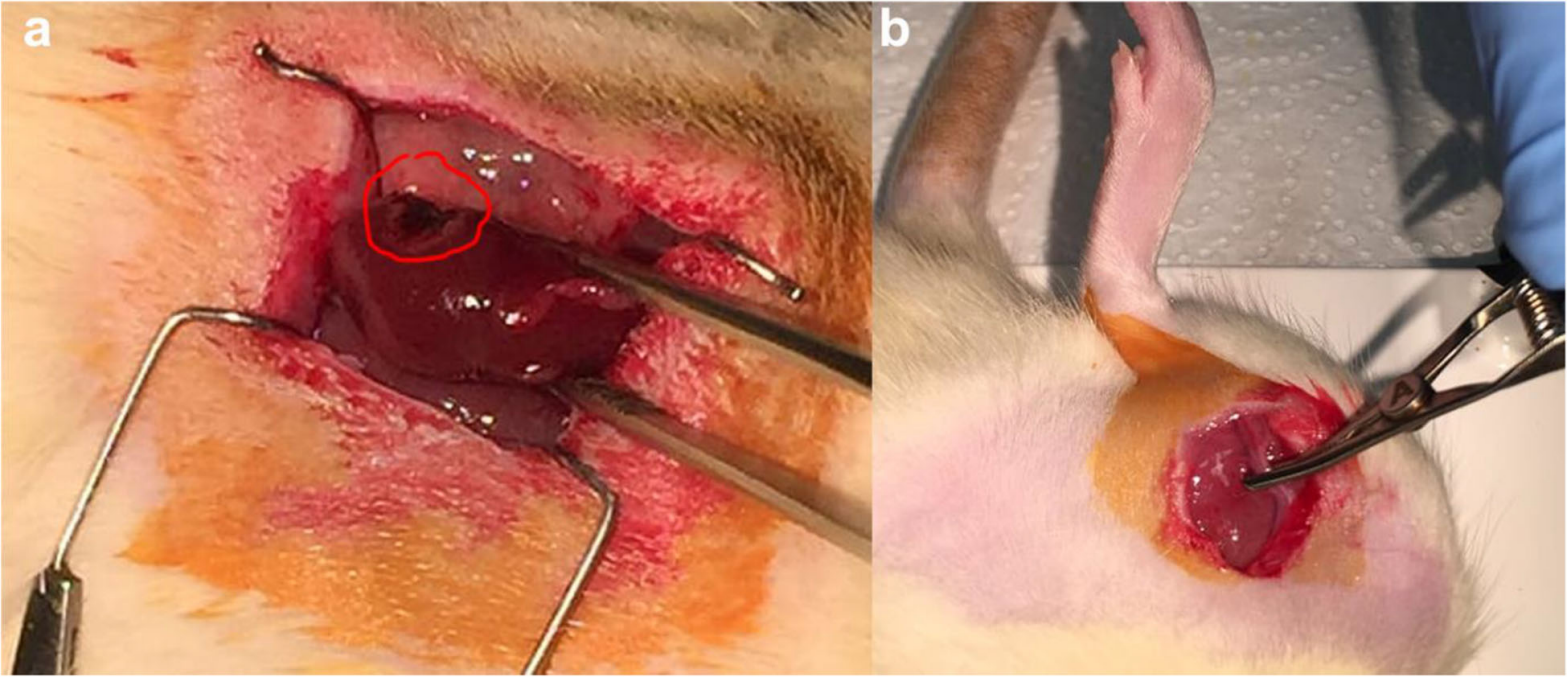
**a** Macroscopic view of the left kidney s/p PN. Red-encircled area is showing the resultant parenchymal defect. **b** Clamping phase of the RIPC cycle. Femoral blood flow (artery and vein) was temporarily interrupted by bulldog clamp

Anesthesia was induced by intraperitoneal injection of Ketamine (70–100 mg/kg) and Xylazine (5–10 mg/kg). This allowed us an operating time of maximum 90 min which included the surgical intervention itself (skin-to-skin) and optimal recovery from anesthesia. Surgeries were performed transperitoneally through a flank incision under aseptic conditions, right nephrectomy was performed in all groups except the sham group. Three days later, PN was performed in the PN and RIPC + PN groups. PN denoted wedge resection of a left renal parenchymal island with a scalpel under warm-ischemic conditions (Fig. 1a). Mean excised kidney tissue volume was 2 mm^3^. In the RIPC + PN group, RIPC preceded PN by 30 min and was employed via sequential clamping / declamping of the femoral artery/vein complex, which was repeated 4 times, each cycle lasting 1 min (Fig. 1b). Warm ischemia was constituted by en-bloc clamping of the renal pedicle for a mean duration of 213 ± 67 s. Renal vessels were freed after securing hemostasis by selective probe electrocauterization of the bleeding spots in the tumor bed. All of the surgical interventions were performed on a heating plate to prevent hypothermia. The sham group underwent laparotomy twice (on days 1 and 3).

**Fig. 2:**
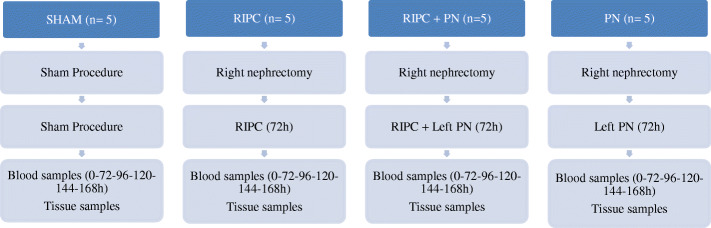
The study protocol: Four groups, each with 5 Wistar albino rats, were constructed; RIPC + PN, PN, RIPC, and sham. Right nephrectomy was performed in all groups except the sham group. Three days later, PN was performed in the PN and RIPC + PN groups. PN was performed under warm ischemia following RIPC. Blood samples were collected on multiple occasions until euthanasia on day 7

Blood samples were drawn from the tail vein, as depicted in the following timeline; at the beginning of the experiment prior to the right nephrectomy, on the 3rd day of the experiment prior to RIPC and/or PN and every 24 h thereafter, until euthanasia on day 7 by cervical dislocation under general anesthesia induced by intraperitoneal injection of Ketamine (70–100 mg/kg) and Xylazine (5–10 mg/kg) (Fig. 2) [5]. Following euthanasia, left kidneys were extracted and cut in half along the sagittal axis. One half was put into 10% buffered formalin and submitted to pathology lab for histological analysis. Remaining kidney tissue and blood samples were stored at − 80 °C for biochemical investigations. Previously excised right kidney specimens were treated in the same manner. Weight measurement and assessment of nutritional and hydration status were conducted at each blood draw session.

### Immunoassays of blood-based biomarkers

Neutrophil gelatinase-associated lipocalin (NGAL) and Kidney Injury Molecule-1 (KIM-1) levels in serum samples and kidney tissue specimens were determined by Sandwich-ELISA using commercial kits (Boster, Pleasanton, USA). Limits of detection (LOD) were, < 10 pg/ml and < 2.0 pg/ml for the NGAL and KIM-1, respectively.

Sandwich-ELISA commercial kits were used to determine the serum and kidney tissue levels of IL-33 (R&D Systems, Minneapolis, USA) and aldose reductase (MyBiosource, San Diego, USA). LODs were, 2.8 pg/ml and < 0.19 ng/ml for the IL-33 and aldose reductase, respectively.

Serum creatinine levels were measured with Creatinine Colorimetric Assay Kit (Cayman, Michigan, USA) based on Jaffe’s reaction. LOD was 0.1 mg/dl for the creatinine assay.

All parameters measured with ELISA were studied in duplicate.

Kidney tissues were homogenized in 100 mmol/L phosphate buffer (pH: 7.4) containing sodium azide (0.05%) for 1 min on ice and then centrifuged at 20.000 g at + 4 °C for 15 min and supernatants were obtained.

### Histopathological examination

**Fig. 3:**
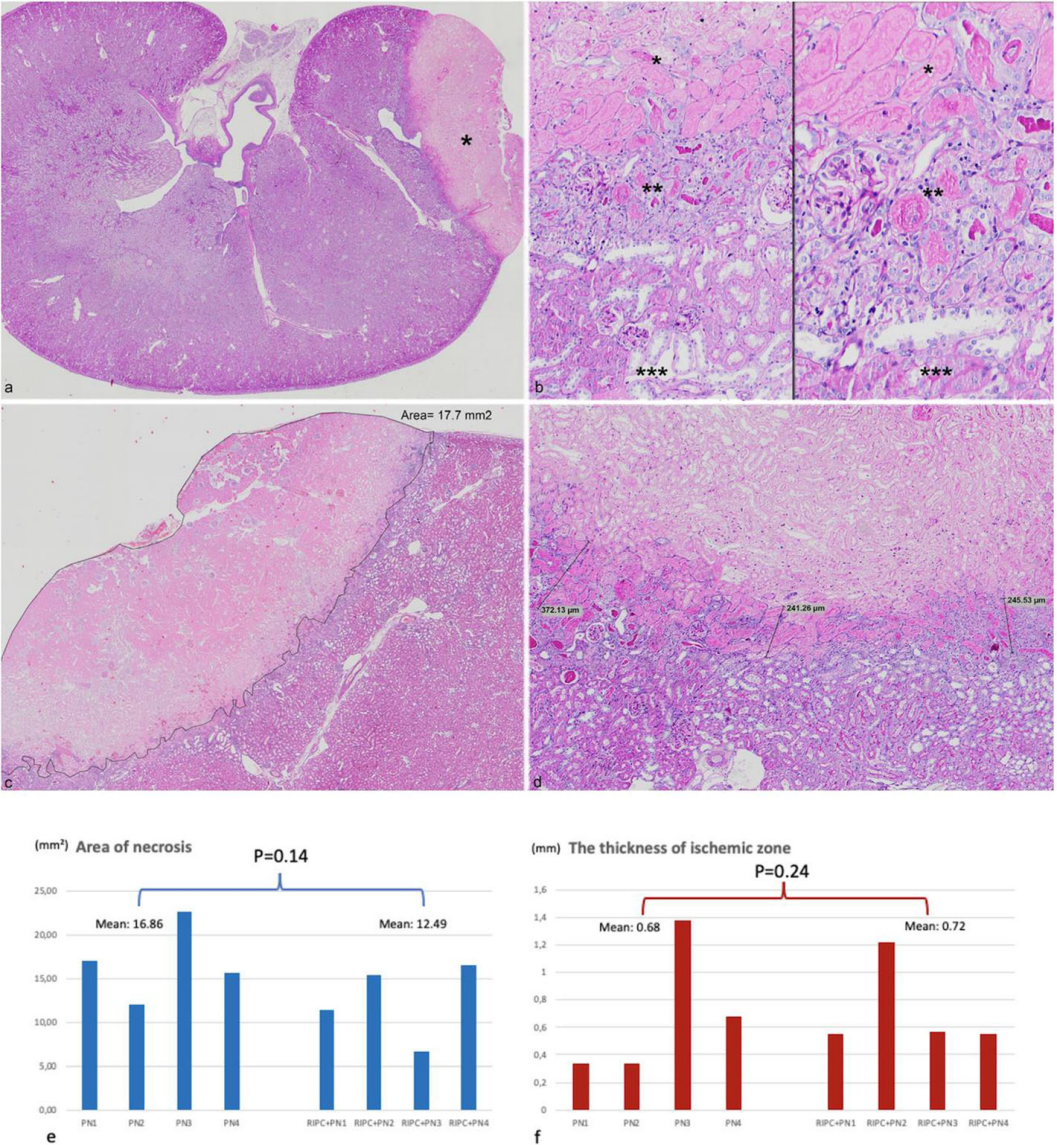
**a** One of the rat kidneys in PN group showing focal infarct (depicted by *) at the resection bed (H-E x 15). **b** Zone of severe ischemia (depicted by **) between regions of infarct (depicted by *) and unaffected renal parenchyma (depicted by ***) (Left: PAS x 100; Right: PAS x 200). **c** and **d** Calculations done by the image analysis program. Panel **C** shows the encircled and measured area of necrosis, while panel **D** demonstrates the thickness readings of ischemic zone through the perpendicular lines drawn 1 mm apart throughout the entire lesion (A: H-E x 30; B: PAS x 90). **e** Area of necrosis in PN and RIPC + PN groups (each blue bar indicating a rat belonging to the groups being compared, p= 0.14). **f** The thickness of ischemic zone in PN and RIPC + PN groups (each red bar indicating a rat belonging to the groups being compared, p= 0.24)

Following overnight fixation in 10% formalin at room temperature, kidney tissues were subjected to routine paraffin embedding procedure. Five sections of 2 μm thick and at 5 μm intervals were obtained from the paraffin blocks. Initial slides were stained with hematoxylin and eosin (H&E) on Sakura Tissue-Tek Prisma automated slide stainer (Nagano, Japan). Remaining 4 were used for additional histochemical stainings with Jones’ methenamine silver (JMS) and periodic acid-Schiff (PAS) methods in order to provide a better assessment of morphology and the extent of histological changes. All light microscopic evaluations were carried out using Olympus BX53 optical microscope. Areas of necrosis and thickness of the zone of severe ischemia were measured for comparison between groups. Measurements were accomplished on digital slides scanned at 40X by Philips IntelliSite Ultra Fast Scanner NOCTN442 (Amsterdam, Netherlands) using the drawing line and area measurement tools of the Image Management System Software (version 3.3.1) (Royal Philips Healthcare, Amsterdam, Netherlands) (Fig. 3c-d). Five μm apart 5 slides (4 special stains and 1 H&E) from each kidney were scanned, area and thickness measurements were done in each section, and average numbers were obtained for each rat.

The hydropic degeneration of the tubular epithelium, single cell tubular necrosis, cast formations and focal interstitial inflammation were scored from 0 to 2 taking into account the frequency of the lesions as 0, none; 1, rare; and 2, common (presence of at least one group of tubules with the lesion on 1 mm^2^ of area).

### Immunohistochemistry

An immunohistochemical staining for Ki-67 was performed to evaluate the regenerative/proliferative activity of the tubular epithelial cells. The BenchMark Ultra automated staining platform (Ventana Medical System, Inc., USA) was used for this purpose. Briefly, the tissue sections from the representative paraffin blocks that were cut at 3 μm thickness onto charged slides were deparaffinized with EZ Prep. solution (Ventana medical system, cat. no: 950–102) at 75 °C. Following rehydration through alcohol series and 32 min heat-induced epitope retrieval at 100 °C with an EDTA-based buffer (Cell Conditioner 1, Ventana; cat. no: 950–124), tissue sections were incubated for 32 min with anti-Ki-67 primary antibody (Ventana; pre-diluted; monoclonal rabbit, clone SP6) at 37 °C temperature. Washing between the steps were accomplished by Reaction Buffer (Ventana medical system, cat. no: 950–300). Ultra View Detection kit (Ventana medical system, cat. no: 760–500) was used for the detection of the target protein. The reaction product was visualized with 3, 3′-diaminobenzidine chromogen and counterstaining with hematoxylin was done. Nuclear staining was considered positive. Appropriate staining of the germinal centers of normal tonsil served as a positive control. Ki-67 index was expressed as the percentage of the number of immunostained nuclei among the total number of tubular cell nuclei.

### Statistical analyses

Statistical comparisons of categorical variables were performed using the chi [2]-test. Continuous variables were compared using Student’s t-test or the Wilcoxon rank sum test depending on whether the data was normally distributed or not. For continuous-paired data, the Wilcoxon signed rank test was used. Longitudinal analyses were performed using linear mixed effect models. All tests were 2-sided, the alpha level for statistical significance was set at 0.05. Statistical analyses were performed with R Statistical Software version 3.6.3 (Foundation for Statistical Computing, Vienna, Austria).

The main reference group was the sham group but in order to account for all possible differences between the study groups and do bidirectional crosscheck, each group has been used once as the reference group.

## Results

In the pilot study, we had no subject loss, but in the actual study one of the rats in the PN group died after the first blood draw (before any surgical procedure). As a result, our mortality rate was calculated to be 5%.

At the start of the study, no statistically significant difference was noted between the study groups in terms of mean body weight, with the overall mean value being 312 ± 32 g. Mean weight loss in the RIPC (22 ± 12.55 g) and RIPC + PN (20.8 ± 11.17 g) groups was significantly more than that recorded in the sham group (1.2 ± 11.97 g) (*p* < 0.001).

The intra- and inter-coefficient of variabilities (CV) were 4.5 and 6.1% for NGAL, 4.2 and 6.2% for KIM-1, 5.3% and 5.0 for IL-33, ≤8.0% and ≤ 10.0% for aldose reductase, were 6.4 and 4.6% for creatinine, respectively.

**Fig. 4:**
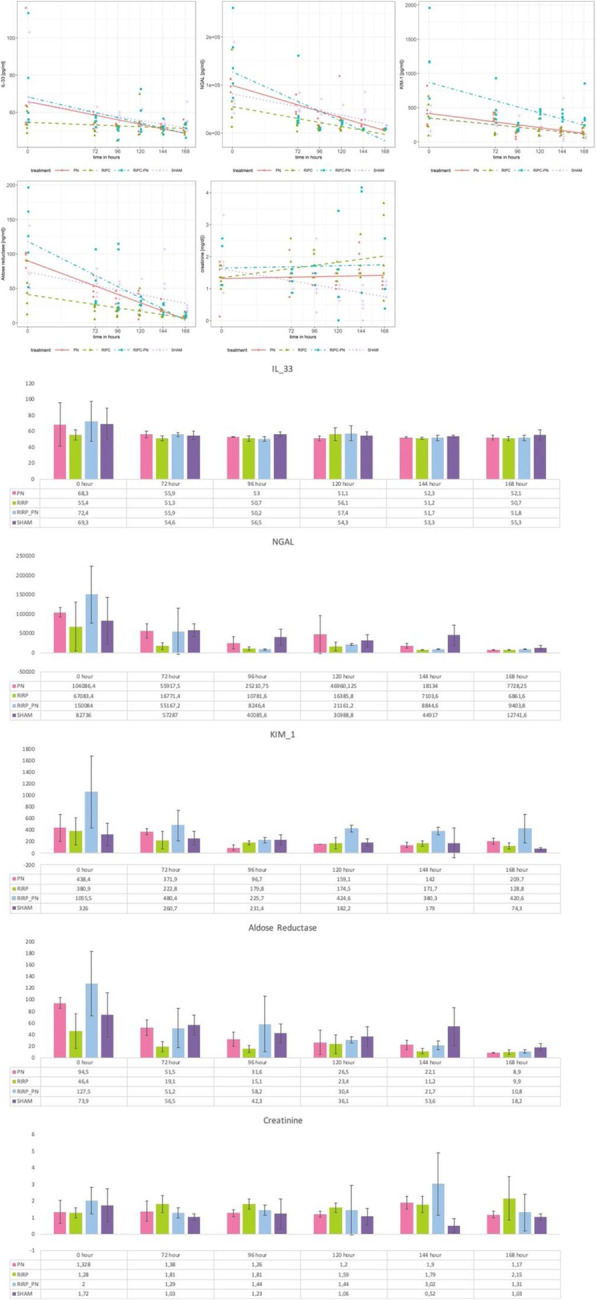
Changes in IL-33, NGAL, KIM-1, aldose reductase, and creatinine values with respect to time across study groups

Regarding IL-33 values, there was no statistically significant difference between the groups. However, mean IL-33 value of the RIPC + PN group was significantly lower than that of the RIPC group (*p* < 0.05, Fig. 4). Likewise, the reduction detected in mean NGAL values of the RIPC + PN group reached statistical significance when compared to the sham and RIPC groups (*p* < 0.01, Fig. 4). Mean values of KIM-1 and aldose reductase were significantly lower in the RIPC + PN group with respect to that of the sham group (*p* < 0.05 and *p* < 0.001, respectively, Fig. 4). In addition, there was a statistically significant difference between the sham and PN groups in terms of the aldose reductase level (p < 0.05, Fig. 4). Creatinine levels demonstrated an increase along the course of the study in all groups when compared with the trend noted in the sham group (Fig. 4). However, this difference was significantly different only between the RIPC and sham groups (*p* < 0.05).

The tissue levels of the biomarkers measured in the left and right kidney specimens exhibited insignificant differences across study groups. In order to control for the effect of the experimental procedures as a whole, we also measured the difference between the right and left kidney specimens in terms of biomarker levels, which similarly revealed statistically insignificant results.

Microscopic examination showed ischemic necrosis (i.e. infarct) in the RIPC + PN and PN groups, at the area of PN (Fig. 3a). There was also a severe ischemic zone between the necrotic area and viable kidney tissue (Fig. 3b).

Regarding the outcome of morphometric analyses; area of total infarct, seen at the resection bed of PN, was larger in the PN group (mean: 16.86 mm^2^) when compared to the RIPC + PN group (mean: 12.49 mm^2^), yet the difference did not reach the level of significance (*p* > 0.05). Mean width of the transitional ischemic zone, the area between the infarcted and viable renal tissue, was similar in both groups, 0.72 mm and 0.68 mm in the RIPC + PN and PN sets, respectively (p > 0.05). Ki-67 proliferative index was 2% in both groups.

No difference was detected in terms of hydropic degeneration of tubular epithelium, single cell necrosis, cast formations or interstitial inflammation between study groups.

## Discussion

The main functional goals of PN are to preserve as much normal tissue as possible without violating oncological principles and to avoid prolonged warm ischemia. Despite all the efforts, a functional decline in the range of 20% is anticipated early after PN. The importance of parenchymal preservation is more pronounced in the setting of imperative or absolute PNs. A retrospective study involving 360 patients with solitary kidneys showed a 6% increase in the incidence of de-novo severe chronic kidney disease (CKD) with each additional minute of warm ischemia. CKD risk was even greater for renal masses with complex morphometric features which would necessitate longer on-clamp duration for a proper PN. In addition, CKD has been shown to be a strong predictor of cardiovascular events and associated mortality in the long run [18, 19].

Several different technical modifications were introduced in an effort to shorten the duration and minimize the functional consequences of warm ischemia time (WIT) during PN, including early unclamping, renal hypothermia, segmental artery clamping and zero-ischemia technique [20–23]. However, data regarding their actual influence on functional and perioperative outcomes is conflicting. Early hilar unclamping was found to be associated with increased blood loss and longer operative duration when compared to the standard approach [24]. Studies about intraoperative cold ischemia showed inconsistent results, with marginal functional benefits only in diabetic and hypertensive patients [25]. The zero-ischemia technique, which involves meticulous dissection of the renal vasculature, was found to be too laborious from the technical standpoint which limited its application to high-volume surgeons.

The need for a simple, effective and reproducible method which could augment the resilience of renal parenchyma against the ischemic insult related to the WIT of PN is currently unmet. RIPC may serve well to fill this gap as it was shown to encompass the potential to promote renoprotection in instances which might indirectly endanger kidney function, such as cardiovascular surgery. Repetition of brief ischemia and reperfusion episodes remotely may result in enhanced tolerance of the kidney to the anticipated, subsequent ischemic damage [26]. This concept was proven by Huang et al., who became the first to demonstrate that remote myocardial preconditioning not only decreased the myocardial infarct area, but also reduced the severity of kidney injury [26]. However, subsequent human and small-animal studies, concentrating on the relationship between RIPC and reversal of acute kidney injury (AKI), revealed somewhat disappointing results [27, 28]. Bedir et al. tested the renoprotective value of RIPC in a porcine solitary kidney model [11]. Their modified study design, which included larger kidneys with a potentially better analogy to human counterparts and a solitary kidney model that eliminated the confounding effect of contralateral kidney on functional analysis, was deemed insufficient to disprove the negative outcomes obtained in prior RIPC studies. Nevertheless, using serum creatinine to monitor functional changes in the kidneys that were subjected to RIPC was their major limitation, as creatinine has poor sensitivity in detecting AKI [29]. Huang et al. tried to overcome the drawbacks of creatinine-based assessment by measuring urinary retinol binding protein levels as a measure of glomerular filtration rate [10]. In their study involving 82 patients, they showed that transient lower limb ischemia reduced renal impairment in the short-term but failed to provide a similar benefit in the long run, despite a positive but statistically insignificant trend in favor of RIPC. However, their outcomes would have been more supportive of RIPC, should they limited their analysis to patients with solitary kidney, which is hard to implement and standardize in a study involving human subjects.

As expected, creatinine levels showed a consistent rise in all groups except the sham group. When compared to the trend observed in the sham group, serum levels of the other biomarkers paradoxically declined along the time course of the study. Inappropriate timing of RIPC and/or PN or the inadequacy of the time interval spent between right nephrectomy and the other experimental procedures might be the underlying reasons for this controversy. We could have shown a more profound protective effect of RIPC if we had proceeded with PN after a longer period of time, allowing the study subjects to show sufficient reaction to the intervention being tested [30]. However, the same trend was also evident for the groups who did not undergo RIPC. Moreover, conducting the pre- and post-intervention follow-up of the rats in the absence of metabolic cage might have influenced their kidney function. Undoubtedly, measuring the urinary levels of these biomarkers, lack of which is a drawback of this study, would have served to strengthen the accuracy of analysis. Lastly, small sample size together with a mortality rate of 5% decreased the power of statistical testing and could be considered as another limitation.

Although we were unable to demonstrate a beneficial impact of RIPC on renal functional preservation from the biomarker standpoint, we have observed a possible hint of its renoprotective effect on histological grounds. This was reflected by a slightly reduced mean infarct area in the RIPC + PN group when compared to the PN group alone. On the other hand, the zone of ischemia, the region between infarcted and unaffected kidney parenchyma was thicker in the former animals which could be explained due to the area protected from complete necrosis being added to ischemic zone. However, these alterations were not found significant statistically. Both groups had similar regeneration capacity as noted by the same Ki-67 proliferative indices.

## Conclusion

According to the results of this experimental study, RIPC failed to counteract the renal functional consequences of PN under warm ischemia in a solitary kidney model. Similarly, benefits at the tissue level were vague and did not appear significant. Our cumulative results via biochemical and histological analyses were not concordant with the renoprotective effect of RIPC.

## Data Availability

All data generated or analyzed during this study are included in this published article. The raw data can be found on https://osf.io/3h4p6/?view_only=8fa3e35c908741bd8ae4cef4dac13951.

## Abbreviations

IPC: Ischemic preconditioning
RIPC: Remote ischemic preconditioning
PN: Partial nephrectomy
KIM-1: Kidney injury molecule
IL: Interleukin
NGAL: Neutrophil Gelatinase-Associated Lipocalin
AKI: Acute kidney injury
WIT: Warm ischemia time
